# Serum carotenoids are strongly associated with dermal carotenoids but not self-reported fruit and vegetable intake among overweight and obese women

**DOI:** 10.1186/s12966-019-0869-3

**Published:** 2019-11-12

**Authors:** Emily H. Morgan, Meredith L. Graham, Grace A. Marshall, Karla L. Hanson, Rebecca A. Seguin-Fowler

**Affiliations:** 1 Department of Nutrition and Food Sciences, University of Vermont, Burlington, VT 05405 USA; 2 Division of Nutritional Sciences, Cornell University, Ithaca, NY 14853 USA; 3 Texas A&M AgriLife Research and Department of Nutrition and Food Science, Texas A&M University, Agriculture and Life Sciences Building, 600 John Kimbrough Boulevard, Suite 512, College Station, TX 77843-2142 USA

**Keywords:** Fruit, Vegetable, Dietary assessment, Carotenoids, Resonance Raman spectroscopy, ASA24

## Abstract

**Background:**

Accurate assessment of fruit and vegetable intake (FVI) is essential for public health nutrition research and surveillance. Blood carotenoid concentrations are robust biomarkers of FVI, but collecting blood samples typically is not feasible in population-based studies. Understanding how well non-invasive measures compare to blood estimates is important for advancing surveillance and evaluation. The objective of this study was to examine the associations between serum carotenoids and four non-invasive measures of FVI in overweight and obese women.

**Methods:**

This study utilized baseline data from 157 overweight or obese women (95.5% white, mean age 58.56 years ± 9.49 years) enrolled in the Strong Hearts, Healthy Communities randomized trial, including two direct measures of carotenoids and three self-reported measures of FVI. Participants completed a fasting blood draw, dermal carotenoid scans using resonance Raman spectroscopy (RRS), a two-item FVI screener modeled after the American Heart Association’s Life’s Simple 7 “My Life Check” tool (modified AHA tool), the National Cancer Institute’s All-Day Fruit and Vegetable Screener (FVS), multiple 24-h dietary recalls, physical measurements, and demographic and health behavior questions. We analyzed blood for total carotenoids and derived total FVI estimates from self-report tools. We used multivariate linear regression models to examine associations between each non-invasive tool and serum carotenoids under four scenarios analogous to different research contexts in which varying breadths of participant data are available. We also calculated adjusted Pearson’s correlations between serum carotenoids, dermal carotenoids, and the self-reported measures.

**Results:**

Dermal carotenoids were strongly correlated with serum carotenoids (0.71, *P* < 0.00067) and associated with serum carotenoids in all regression models (0.42–0.43, *P* < 0.002). None of the self-reported FVI measures were significantly associated with serum or dermal carotenoids in adjusted regression models or correlation analyses.

**Conclusions:**

Compared to self-reported FVI, we found dermal carotenoids measured by RRS to be a superior method to approximate serum carotenoids among overweight and obese women. More research is needed to investigate these assessment methods in diverse populations.

**Trial registration:**

ClinicalTrials.gov Identifier: NCT02499731, registered July 16, 2015.

## Background

Dietary patterns high in fruit and vegetable intake (FVI) are associated with lower risks for multiple chronic, non-communicable diseases, particularly cardiovascular disease and some cancers [1], and all-cause mortality [2]. In part, the beneficial effects of FVI are thought to be due to their carotenoid content and the modulating effect of carotenoids on oxidative stress and inflammation [3, 4]. A recent systematic review demonstrated strong inverse associations between blood carotenoids and the grouping of cardiometabolic risk factors known as metabolic syndrome [5]. However, the majority of adults in the United States do not consume the recommended amounts of fruits and vegetables [6] and increasing FVI remains an important target for chronic disease risk reduction.

Accurate measurement of FVI is important for public health surveillance, the evaluation of nutrition interventions, and testing hypotheses regarding FVI and health outcomes. Carotenoid concentrations in the blood are considered the best biomarkers of FVI [7, 8]. However, blood samples are invasive and costly to collect and analyze, thereby limiting their use in population-based research. Further, blood carotenoids are less suitable for studies focused on usual FVI, as they are influenced by recent dietary intake, with an estimated carotene half-life of less than 2 weeks [9]. The most common methods to assess diet in larger observational and community-based studies rely on self-reported intake tools such as 24-h (24-h) dietary recalls, food records, food frequency questionnaires (FFQs), and dietary screeners [10–12]. Dietary recalls and food records collect greater detail than FFQs and dietary screeners, including daily macronutrient and micronutrient intake values, but multiple days of data are required to represent usual intake, thus putting a considerable burden on participants. Well-documented weaknesses of self-reported dietary intake include recall or response bias for reasons related to memory, knowledge, and perceived social desirability; failure to capture seasonal variation in diet; the possibility that participants may alter eating behaviors in anticipation of reporting; and within-person random error due to day-to-day variation in food and drinks consumed [10–12].

Dermal carotenoid levels measured using innovative, non-invasive technologies offer a promising alternative assessment method. One such technique, resonance Raman spectroscopy (RRS), utilizes a small probe with a laser at a blue wavelength to measure total dermal carotenoid levels [13]. Dermal scanning is fast (about 30 s per measurement) [14], requires minimal technical expertise, and can be conducted in field settings using portable scanners. Previous research has found dermal carotenoid levels measured by RRS to be reproducible [15] and valid compared with blood concentrations [15–19]. The half-life of carotenoids in the skin appears to be longer than that in blood, with the skin acting as a longer-term reservoir for carotenoids [16, 17, 20].

There is a continued need for evaluation of how well noninvasive measures of FVI compare to blood biomarkers like carotenoids to help researchers evaluate the appropriateness of different measurement techniques for estimating FVI in settings in which drawing blood for dietary assessment may be inappropriate or impractical. This is particularly important in population subgroups at elevated risk of diet-related disease. Among participants in the Strong Hearts, Healthy Communities (SHHC) trial [21, 22], we had the opportunity to examine the associations between serum carotenoids and dermal carotenoids measured using RRS and three self-reported measures of FVI for the first time in a sample of overweight and obese rural women. We hypothesized that serum carotenoids would be associated with each non-invasive measurement, with the strongest association between serum and dermal carotenoids. We examined associations under four scenarios analogous to different research contexts in which investigators would have access to different breadths of participant data. Specifically, we sought to examine associations under a field-based scenario, a non-clinical research scenario, and a clinical research scenario.

## Methods

SHHC was a randomized, controlled trial aimed at evaluating the effectiveness of a community-based multilevel cardiovascular disease prevention program for midlife and older women living in medically underserved rural communities (ClinicalTrials.gov NCT02499731). Complete details regarding the study design and methods [22] and trial findings [21] are detailed elsewhere. The study was approved by the Institutional Review Boards at Cornell University (file #1402004505) and Bassett Medical Center (file #2022). In this article, we use cross-sectional baseline data from the trial. We purposefully administered multiple measures of FVI and carotenoids to support their evaluation and comparison.

### Study population

A total of 194 women aged 41–84 living in 16 government-designated medically underserved rural communities in Montana and New York were enrolled in the trial. Inclusion criteria were age 40+ years, BMI ≥ 25, currently sedentary, English-speaking, physician’s approval to participate, and willingness to be randomized to one of two community programs. Exclusion criteria included blood pressure > 100 (diastolic) or > 160 (systolic), resting heart rate < 60 or > 100, cognitive impairment, or lack of willingness or ability to complete online questionnaires. Baseline data were collected September through December 2015. For each woman, all baseline data used in analyses were collected within a six-week time period from consent.

### Sociodemographic and general health information

Participants completed an online questionnaire that collected the following data: sociodemographic characteristics including age, race, employment status, and income; self-reported height and weight, which were used to calculate self-reported BMI; and health behaviors including smoking status, use of lipid-lowering medication, and whether they had been diagnosed with any of the following chronic conditions: heart disease, diabetes, high blood cholesterol, or hypertension. The content of the questionnaire was derived from national surveys and established public health survey instruments and has been described previously [17].

### Anthropometric measurements

Trained research staff collected physical measurements at a baseline assessment prior to randomization [22]. Baseline assessments were held in community spaces such as churches and county office buildings. Participants were clothed but without shoes and socks for height and weight measurements. Height was measured using a free-standing stadiometer and weight was measured with the Omron Full Body Sensor Body Composition Monitor and Scale. Measurements were taken in duplicate unless specified criteria for the proximity of measurements were not met by the first two (height differed by ≥3.175 mm [0.125 in]; weight measurements differed by ≥0.227 kg [0.5 lbs]); in this case, a third measurement was taken. Mean height and mean weight measurements were used to calculate measured BMI.

### Blood collection and laboratory analyses

Fasting blood specimens were collected by venipuncture by phlebotomists and registered nurses in community locations behind privacy screens. One serum separator tube (10 mL) was labeled with participant identification number, centrifuged at 3200 pm, and shipped in a thermal insulated container with refrigerant packs to Quest Diagnostics (Denver, CO) or Bassett Healthcare (Cooperstown, NY) for the lipid analyses, including total cholesterol (mg/dL) and total triglycerides (mg/dL). Bassett Healthcare used a Dimension Vista 1500 Analyzer (Siemens Healthcare Diagnostics). The limit of detection is 2 mg/dL for the triglyceride assay and 50 mg/dL for the cholesterol assay. Quest Diagnostics ran the HealthScreen-TSH/Lipid-DirLDL/Ferr (Test number: 336616) using chemiluminescent immunoassays. The limit of quantification is 6.79 mg/dL for the triglyceride assay and 21.3 mg/dL for the cholesterol assay. Both methods are enzymatic.

Another labeled serum separator tube was shipped to Cornell University where the sample was aliquoted, stored at − 80 °C, and subsequently shipped to Craft Technologies Inc. (Wilson, NC, USA) for analyses of fractionated carotenoids by HPLC with multi-wavelength photodiode-array detection. Carotenoid analyses used 150 μL of serum and a detection limit of 0.005 μg/mL. Calibration of the procedure involved use of neat standards for each carotenoid and an internal standard to adjust for extraction efficiency. Linear calibration curves were generated for each analyte. The laboratory provided results for total carotenoids (μg/g), which were used in the present analyses. We paid $63 per sample for the serum carotenoid analysis.

### Dermal carotenoid measurements

Dermal carotenoids were measured by research staff at the community-based baseline assessments using the Pharmanex© Biophotonic Scanner S3 (NuSkin Enterprises, Provo, UT, USA), which uses the RRS technology. Market pricing is not available for this product, but another portable optical device for measuring dermal carotenoids, the Veggie Meter, can be purchased for $15,000. The Pharmanex© Biophotonic Scanner S3 used in this study uses a blue laser light (λ = 478 nm) to excite dermal carotenoids at a specific site on the palm of the hand and then calculates a dermal carotenoid score based on the detection of scattered green light (λ = 518 nm). The scanner was calibrated each day prior to use. RRS scanner readings can range from 0 to 90,000; higher scores indicate higher concentrations of carotenoid molecules at the measurement site. The scanner does not generate separate scores for individual carotenoids due to overlap in the absorption spectra of all carotenoids. Each participant was scanned twice. If the scores differed by > 3000 units, a third scan was conducted. The mean of the two or three scores was used in this analysis.

### Self-reported dietary intake measurements

#### Modified American Heart Association questions

Participants were asked two questions about their typical daily consumption of fruits and vegetables adapted from the American Heart Association’s Life’s Simple 7 “My Life Check” tool (hereafter referred to as the modified AHA tool) as part of the online questionnaire. Thirteen response options for each question ranged from zero to six cups in half cup increments, and relevant portion examples were provided for each question. Fruits and vegetables were summed into total cups for this analysis.

#### All-day fruit and vegetable screener

Participants also completed the validated All-Day Fruit and Vegetable Screener (FVS) [23] developed by the National Cancer Institute (NCI) as part of the online questionnaire. The FVS is a 19-item instrument that collects data on fruits and vegetables consumed in the past month. Closed-ended questions query frequency of consumption (ten response categories ranging from never to five or more times per day) and portion size (four response categories with options dependent on the food item). Total FVI estimates for the present analysis were calculated excluding potatoes and beans, which contribute few dietary carotenoids [24].

#### Automated self-administered 24-hour recalls

Participants were asked to complete seven dietary recalls using the validated Automated Self-Administered 24-Hour (ASA24) 2014 web-based tool developed by the NCI [25–27]. The system queried all foods and beverages consumed on the previous day. All items reported are mapped to the USDA MyPyramid Equivalents Database. Following data collection, food group variables were updated into the more recent Food Patterns Equivalents Database using the SAS program provided on the NCI website [28]. If a participant recorded that she consumed alcohol on any of her dietary recalls, she was categorized as consuming alcohol. In addition to the standard module, participants were asked to complete an auxiliary module on supplement intake, which was used to categorize whether participants used any dietary supplements, and carotenoid-containing dietary supplements in particular. Intakes of total energy, fat, and FVI were calculated as the mean kilocalories (kcal), grams of fat, and cup-equivalents of fruit and vegetables consumed across all recalls. To generate total dietary carotenoids (μg), average intakes of α-carotene, β-carotene, β-cryptoxanthin, lycopene, lutein, and zeaxanthin were summed. As with the FVS, total FVI estimates were calculated excluding potatoes and beans.

### Statistical analysis

All 24-h recalls were examined and excluded if they were likely to be missing data (marked incomplete and included < 600 kcal or marked complete and included < 100 kcal) or identified as implausible (marked complete but included > 6600 kcal, i.e. three times the estimated energy requirement for a physically active woman aged 31–60 years). For each participant with at least two valid dietary recalls, mean energy, fat, FVI, and total dietary carotenoids were calculated from two to seven valid recalls.

Of the 194 women consented to the SHHC study, we excluded 37 who were missing data for serum carotenoids; dermal carotenoids; FVI from the modified AHA tool, FVS, or ASA24; and/or any of the covariates included in our final models (as shown below).

Descriptive statistics were used to summarize the characteristics of the study sample and examine the distribution of values for all measures. Normality was assessed by visually inspecting the distributions of each variable and checking that the skewness and kurtosis were between ±2.0. Serum cholesterol (mg/dL), total serum carotenoids (μg/g), dermal carotenoid score, FVS estimates (cup-equivalents), 24-h recall FVI estimates (cup-equivalents), and 24-h recall total dietary carotenoids (μg) had a kurtosis greater than 2.0. To improve normality, these variables were transformed using ln(x + 1) for analyses (but not descriptive reporting).

Using linear regression we examined whether each of the non-invasive (i.e. dermal or self-report) measures was associated with serum carotenoids. Following the construction of unadjusted models, we used multiple regression analysis to model the relationships under three hypothetical research scenarios: a *field-based scenario* (population-based research with minimal contact between the research team and participants), a *non-clinical research scenario* (population-based research with moderate contact between the research team and participants), and a *clinical research scenario* (research in a healthcare setting with routine contact between the research team and participants). We considered covariates that were hypothesized or have been found by others to influence blood carotenoids [9, 13, 29–31]. Prior studies have shown smoking, alcohol use, inflammation and related chronic diseases, adiposity, and sun exposure to be associated with lower carotenoid concentrations [9, 13]. Because carotenoids circulate in lipoproteins, an individual’s lipid profile, use of medications that impact blood lipids (e.g. cholesterol-lowering drugs), or total energy intake may affect carotenoid concentrations [9]. Age and skin pigmentation also have been suggested as possible determinants of carotenoid concentrations, but evidence is limited and inconsistent [9, 13]. Measurable increases in blood and tissue carotenoids following carotenoid supplementation are well documented [9, 13]. In initial versions of the models for this study, we controlled for race, age in years, BMI (self-reported and measured), state (capturing state-specific characteristics and a proxy for sun exposure), smoking, alcohol use, chronic disease diagnosis, any supplement use, carotenoid-containing supplement use, use of a lipid-lowering medication, total energy intake, a measure of lipids (either self-reported fat intake generated from the ASA24 as a measure of dietary lipids or log serum cholesterol as a measure of circulating lipids), and serum triglycerides. We retained state, age in years, BMI, and the measures of lipids in our final models, either because of the strength of the evidence related to their influence on carotenoid concentrations or because they meaningfully changed the regression results.

In the *field-based scenario*, we assumed only self-report data would be possible and therefore incorporated state, age, and self-reported BMI as covariates. In the *non-clinical research scenario*, we assumed measurements could be taken, but collection of blood data would not be possible and therefore included state, age, measured BMI, and self-reported fat intake as covariates. In the *clinical research scenario*, we incorporated state, age, measured BMI, and log serum cholesterol. We determined the best model by finding the smallest raw Akaike information criterion (AIC) value and then calculated ΔAIC by subtracting the smallest AIC from the AIC of each other model. We considered ΔAIC ≤2 to indicate equivalency with the best model and ΔAIC > 10 to indicate essentially no evidence that the model was as good as the best model [32]. Bonferroni correction was applied to tests of coefficients across the five models such that *P* < 0.01 denotes 95% confidence and *P* < 0.002 denotes 99% confidence. The first four models correspond to dermal carotenoids and the three self-report measures of FVI, and the fifth model corresponds to total dietary carotenoids, considered as a sensitivity analysis.

Pearson’s partial correlations between serum carotenoids and the non-invasive measures were performed adjusting for variables in the *clinical research scenario* (state, age, measured BMI, and log serum cholesterol). Daily variability in dietary intake introduces within-person variance that can attenuate correlations between FVI measured by multiple 24-h recalls and measures of usual intake [12]. To correct for this, we de-attenuated the correlations including 24-h recall data. We considered correlations < 0.2 as weak, 0.2–0.6 as moderate, and > 0.6 as strong [33]. Bonferroni correction was applied to the fifteen tests of significance for the adjusted correlations (*P* < 0.0033 denotes 95% confidence and *P* < 0.00067 denotes 99% confidence). De-attenuated correlations were calculated by multiplying the Pearson’s correlation coefficient by an attenuation factor [12]. The attenuation factor was calculated using the equation $\sqrt{1+\left(\frac{{s_w}^2}{{s_b}^2}\right)/n}$, where *s*_*w*_^2^ is the within-person variance and *s*_*b*_^2^ is the between-person variance of the log transformed 24-h recall variable. In this case, *n* is the number of valid 24-h recalls used to create participant mean values. However, since all participants did not complete the same number of recalls, the median of seven was used.

Data were analyzed using SPSS version 25 (IBM Corp., Armonk, NY, USA) and SAS version 9.4 (SAS Institute, Inc., Cary, NC, USA).

## Results

Participants were predominantly white, non-smokers, and employed; mean age was 59 years (Table 1). Mean measured and self-reported BMI were both approximately 35 (class 2 obesity). Women reported consuming on average 2.33–3.99 cups of fruit and vegetables per day, with the 24-h recalls estimating the lowest FVI and the FVS estimating the highest. Mean daily energy intake was approximately 1800 kcal, mean dermal carotenoid score was approximately 34,100, and mean total dietary carotenoids was nearly 10,000 μg.

**Table 1:** Characteristics of study participants (*n* = 157)

	Mean or Number	SD or %
Age (years)	58.56	9.49
Race		
White	150	95.5
Other	6	3.8
Not reported	1	0.6
Employment status		
Employed	106	67.5
Unemployed	5	3.2
Homemaker	9	5.7
Retired	36	22.9
Not reported	1	0.6
Household income		
< $25,000	30	19.1
$25,000 to < $50,000	42	26.8
$50,000 to < $75,000	34	21.7
≥ $75,000	41	26.1
Not reported	10	6.4
Smoking status		
Current smoker	6	3.8
Former smoker	41	26.1
Never smoker	110	70.1
Alcohol use		
Yes	73	46.5
No	84	53.5
Dietary supplement use		
Yes	113	72.0
No	44	28.0
Carotenoid-containing dietary supplement use		
Yes	46	29.3
No	111	70.7
Lipid-lowering medication use		
Yes	40	25.5
No	38	24.2
Not reported	79	50.3
Chronic disease diagnosis ^1^		
Yes	113	72.0
No	44	28.0
Anthropometric characteristics		
Measured BMI	35.72	6.48
Self-reported BMI	35.30	6.50
Dermal carotenoid score	34,067.94	10,454.01
Blood measurements		
Serum carotenoids (μg/g)	0.85	0.36
Serum cholesterol (mg/dL)	201.78	42.92
Serum triglycerides (mg/dL)	143.85	74.51
Fruit and vegetable intake (cups/day)		
Modified AHA tool	2.80	1.47
FVS ^2^	3.99	2.68
24-h recall ^2^	2.33	1.38
Dietary characteristics		
Energy intake (kcal/d)	1801.79	504.20
Total fat intake (g/d)	78.39	27.38
Total dietary carotenoids measured by 24-h recall (μg)	9971.89	6225.30

^1^Includes diagnoses of hypertension, high cholesterol, heart disease, diabetes, arthritis, cancer, and kidney disease

^2^Estimate excludes potatoes and beans

In unadjusted regression models, dermal carotenoids and FVI estimated by the modified AHA tool were significantly associated with serum carotenoids (Table 2). Only the association between dermal and serum carotenoids remained significant in adjusted regression models (*P* < 0.002), with the model for the *clinical research scenario* explaining 61% of the variability in serum carotenoids. Total dietary carotenoids were not associated with serum carotenoids in any of the models (data not shown). In all cases, the AIC values indicated the *clinical research scenario* model to be the best model. Using ∆AIC, we determined the *field-based* and *non-clinical research scenario* models to be equivalent to each other and stronger than the unadjusted model.

**Table 2:** Change in log serum carotenoids per unit change in log dermal carotenoids and self-reported FVI ^1,2^

	Unadjusted Model		Field-based Scenario Model		Non-clinical Research Scenario Model		Clinical Research Scenario Model	
	β	SE	β	SE	β	SE	β	SE
Log dermal carotenoid score	0.43**	0.04	0.43**	0.04	0.43**	0.04	0.42**	0.03
State			0.08**	0.02	0.08**	0.02	0.05	0.02
Age (years)			0.00	0.00	0.00*	0.00	0.00	0.00
Self-reported BMI			0.00	0.00				
Measured BMI					0.00*	0.00	0.00	0.00
Self-reported fat intake (g/day)					0.00	0.00		
Log serum cholesterol (mg/dL)							0.26**	0.05
R^2^	0.44		0.54		0.55		0.61	
ΔAIC	49.35		24.74		24.43		0.00	
Modified AHA tool FVI (cups/day)	0.03*	0.01	0.02	0.01	0.03	0.01	0.02	0.01
State			0.02	0.03	0.02	0.03	−0.01	0.03
Age (years)			0.00	0.00	0.00	0.00	0.00	0.00
Self-reported BMI			−0.01**	0.00				
Measured BMI					−0.01**	0.00	− 0.01*	0.00
Self-reported fat intake (g/day)					0.00	0.00		
Log serum cholesterol (mg/dL)							0.29**	0.07
R^2^	0.05		0.15		0.17		0.24	
ΔAIC	26.86		14.64		13.84		0.00	
Log FVS FVI (cups/day) ^3^	0.07	0.03	0.06	0.03	0.07	0.03	0.06	0.03
State			0.03	0.03	0.02	0.03	0.00	0.03
Age (years)			0.00	0.00	0.00	0.00	0.00	0.00
Self-reported BMI			−0.01**	0.00				
Measured BMI					−0.01**	0.00	−0.01*	0.00
Self-reported fat intake (g/day)					0.00	0.00		
Log serum cholesterol (mg/dL)							0.29**	0.07
R^2^	0.04		0.15		0.16		0.24	
ΔAIC	28.32		14.63		14.38		0.00	
Log 24-h recall FVI (cups/day) ^3^	0.06	0.04	0.02	0.04	0.02	0.04	0.04	0.04
State			0.03	0.03	0.03	0.03	−0.01	0.03
Age (years)			0.00	0.00	0.00	0.00	0.00	0.00
Self-reported BMI			−0.01**	0.00				
Measured BMI					−0.01**	0.00	−0.01*	0.00
Self-reported fat intake (g/day)					0.00	0.00		
Log serum cholesterol (mg/dL)							0.30**	0.07
R^2^	0.01		0.13		0.14		0.22	
ΔAIC	28.82		15.50		16.19		0.00	

^1^*n* = 157

^2^*FVI* Fruit and vegetable intake, *FVS* National Cancer Institute All-day Fruit and Vegetable Screener, *AHA* American Heart Association

^3^Potatoes and beans removed from dietary FVI measures when possible

Bonferroni corrected significance denoted: * *P* < 0.01 (95% confidence) and ** *P* < 0.002 (99% confidence)

Table 3 presents *clinical research scenario*-adjusted correlations among serum carotenoids and all non-invasive measurements. There was a strong, significant, positive correlation between dermal carotenoids and serum carotenoids (0.71, *P* < 0.00067). No significant correlations were observed between self-reported FVI and serum carotenoids or dermal carotenoids. All self-reported measurements were positively correlated with each other. FVI measured by the modified AHA tool was strongly correlated with FVI estimated by the FVS (0.71, *P* < 0.00067) and moderately correlated with FVI estimated by the 24-h recalls (0.40, *P* < 0.00067). FVI estimated by the FVS and 24-h recalls were moderately correlated (0.47, *P* < 0.00067) with each other. Total dietary carotenoids was correlated with FVI estimated by the FVS and 24-h recalls, but was not correlated with serum carotenoids, dermal carotenoids, or FVI estimated by the modified AHA tool (data not shown). Substantial changes in magnitude were not observed when FVI estimated by the 24-h recalls was de-attenuated (presented below the diagonal on Table 3).

**Table 3:** Pearson’s partial correlations between log serum carotenoids, log dermal carotenoids, and self-reported FVI ^1,2^

	Log serum carotenoids	Log dermal carotenoid score	Modified AHA tool FVI (cups/day)	Log FVS FVI (cups/day)	Log 24-h recall FVI (cups/day)
Log serum carotenoids		0.71*	0.18	0.17	0.09
Log dermal carotenoid score			0.23	0.15	0.15
Modified AHA tool FVI (cups/day)				0.71*	0.40*
Log FVS FVI (cups/day)					0.47*
Log 24-h recall FVI (cups/day) ^3^	0.09	0.16	0.44	0.51	

^1^*n* = 157

^2^*FVI* Fruit and vegetable intake, *FVS* National Cancer Institute All-day Fruit and Vegetable Screener, *AHA* American Heart Association. Adjusted correlations control for the effects of age (years), state, log total serum cholesterol (mg/dL), and BMI (kg/m2)

^3^De-attenuated coefficients for the 24-h recall correlations are presented below the diagonal

Bonferroni corrected significance denoted: * *P* < 0.00067 (99% confidence)

## Discussion

In this study, we found that dermal carotenoid levels measured by RRS were strongly associated with serum carotenoids; whereas, self-reported FVI measured by the two-item modified AHA tool, the validated NCI FVS, and multiple 24-h recalls using the ASA24 were not associated with serum carotenoids. Unlike past work in this area [9, 13, 16–18, 25, 33–36], we focused on overweight and obese women, a group often targeted in chronic disease prevention interventions, and for whom blood carotenoid concentrations tend to be lower [8, 9, 37–41]. The study included high quality serum carotenoid measures and three widely used self-report FVI instruments as well as an innovative and noninvasive technique for measuring dermal carotenoids that is increasingly used for nutrition assessment [42]. Also, we evaluated how different breadths of participant data influenced the findings, and found that, in settings in which measured information about participants is available including measured height and weight and blood cholesterol, the explanatory capabilities of the models were improved by adjustment.

Another novel contribution of our findings relate to the rural, community-based setting for the research. Collecting data in rural areas presents unique research challenges [43, 44]. Difficult field conditions, including long distances between sites, limited post office hours, and poor access to critical research supplies such as dry ice, were overcome to meet operational requirements for collection, management, and shipment of biological samples. Despite these barriers, we found that dermal carotenoid levels were strongly correlated with serum carotenoids and remained significant in regression analyses under all four adjustment models. The observed adjusted correlation coefficient of 0.71 is comparable to previously published studies with adults, which identified unadjusted correlations between RRS-assessed dermal carotenoids and blood carotenoids between 0.61 and 0.82 [15–18]. Compared to previous research, our study population was notably older and unique in that all participants had a BMI ≥ 25 and were sedentary. To the best of our knowledge, this is the first study to examine correlations between blood and dermal carotenoid levels from data collected in rural areas. We found collecting dermal measurements to be feasible and that they resulted in stronger models than self-reported FVI. In fact, participation in our data collection was highest for measurements that involved face-to-face interactions [21, 45]. Thus, our findings add to the growing body of evidence indicating measurement of dermal carotenoids to be a promising alternative for assessing serum carotenoid status in nutrition studies in a variety of community and laboratory settings, including among overweight and obese populations. When used across multiple studies or for large samples, the cost of dermal carotenoid scanning is likely to be less expensive than collecting, shipping, and analyzing serum carotenoid samples. Future studies also should consider collecting cholesterol data for inclusion in adjustment models via simpler blood collection procedures like point-of-care lipid profile testing that relies on a rapid and minimally invasive technique, for example a finger prick. Such tests can simplify blood collection and may increase feasibility at community locations.

Aligning with prior research [23, 25, 46, 47], we found FVI estimated by each self-report instrument to differ, with the FVS estimating the highest intake and the 24-h recalls estimating the lowest. The difference in mean FVI estimated by the FVS and 24-h recalls was 1.66 cups. A study by Yaroch et al. modified the FVS to a 16-item format and found that intake estimates derived from the modified version did not differ from those obtained from 24-h recalls [47]. However, that study also evaluated a two-item FVI screener (similar to the modified AHA questions) and found that the two-item tool overestimated FVI relative to recalls. Correlations among the three self-reported measures of FVI were moderate to strong, significant, and roughly similar in magnitude to those reported in most other studies [23, 34, 46, 47].

We found no significant adjusted correlations between FVI measured by self-report and serum carotenoids. Although not significant in this study, the correlation observed between FVI estimated by the modified AHA tool and serum carotenoids was similar in magnitude to that observed by Resnicow et al. in an unadjusted evaluation of a similar two-item instrument [36]. The non-significant correlation between serum carotenoids and FVI measured by FVS was similar to that identified among women by Greene et al. at one site of their multi-study analysis, but notably lower than that observed at another site [34]. Non-significant correlations observed between serum carotenoids and FVI measured by the 24-h recalls were lower than those found by both Greene et al. 2008 [34] and Resnicow et al. 2000 [36]. An analysis of midlife and older adults by George et al., documented moderate to strong correlations in adjusted models between serum carotenoids and carotenoid intake estimated from three self-reported instruments: a diet history questionnaire, a seventeen-item FVI screener, and de-attenuated 24-h recalls [25]. A key distinction between their work and ours is that they derived estimates of individual carotenoids from each self-reported instrument, while we prioritized evaluation of total FVI given its greater prevalence as a measure in behavioral nutrition research. However, we did examine correlations between total dietary carotenoid intake and the other carotenoid and FVI measurements and found no relationship with either serum or dermal carotenoids. Although previous research has found self-reported FVI to be associated with dermal carotenoid levels [13, 16, 33, 35], we did not find any self-report measure to be correlated with dermal carotenoids in the present study. Given the notable deviations of these findings from previous research, replication studies are needed.

In the United States, over 40% of adults aged 40+ years are now obese [48]. Although there is consistent evidence that blood carotenoids are a viable biomarker for FVI [8, 37], prior research indicates that carotenoid status varies by body composition with greater BMI associated with lower blood carotenoids [8, 9, 37–41]. Previous studies have examined associations between carotenoid intake, blood carotenoid concentrations, and obesity [40, 41]. In a sample of 542 older adults, Vioque et al. observed plasma carotenoids to be positively associated with dietary carotenoids (estimated by FFQ) and negatively associated with BMI [40]. The study also found significant interactions between BMI and selected carotenoids, with weaker correlations between dietary carotenoids and plasma carotenoids observed in overweight and obese adults. Similarly, in a study of 3128 midlife adults, Galan et al. found dietary β-carotene (estimated by multiple 24-h recalls) to be positively associated with serum β-carotene [41]. Among men, the study also observed serum β-carotene levels to be lower in obese participants. Possible mechanisms include obesity-mediating inflammation giving rise to higher expenditure of circulating antioxidant carotenoids, greater adiposity resulting in a higher proportion of ingested carotenoids being absorbed in adipose tissue, or lower FVI [9, 39]. We are not aware of any studies that evaluate associations between dietary carotenoids or FVI, dermal carotenoid status, and weight status in adults. The elevated BMIs of participants in our sample could be one reason for the differences in observed correlations between our study and others. However, of note, the average dermal carotenoid level of our sample was comparable or even slightly higher than what has been documented in prior studies with adults [18, 49, 50].

In addition to heavier body weights in our sample, other possible explanations include varying measurement instruments, definitions of FVI, and covariates [51], as well as the use of an online data collection instrument. For instance, Resnicow et al. [36] examined African American adults enrolled in a healthy eating intervention and Greene et al. [34] drew together data from five independent behavior change studies with distinct samples and goals; neither study focused on overweight and obese women, nor did their analyses adjust for serum cholesterol, which we observed to be significant in all clinical models. Both of these earlier studies were conducted prior to the introduction of the ASA24 and relied on three telephone-based, interviewer-administered recalls using the multiple pass approach. Future investigations should examine whether associations between reported FVI and blood and dermal carotenoids differ between normal weight and overweight and obese individuals, and whether online dietary data collection results in higher levels of misreporting among older adults.

Our findings are likely to be useful for researchers selecting instruments for nutrition research. The median time to complete a recall with the current version of the ASA24 is 24 min, with a respondent’s first recall typically taking two to three minutes longer compared to subsequent recalls [33]. To estimate usual FVI using recommended protocols, multiple recalls are required, which consume hours of participant time [10–12]. This can be compared to several minutes required to complete the FVS or seconds needed to complete a dermal carotenoid scan or to answer the two modified AHA questions. Of course, when data beyond carotenoid status or total FVI is needed, such as when the intent is to compare intake to recommendations, methods that capture daily intake (e.g. multiple 24-h recalls or food records) remain essential to produce a comprehensive picture of an individual’s diet.

Concentrations of carotenoids in the blood and skin depend on a number of factors in addition to FVI, which may explain, in part, why we did not find self-reported FVI to relate to serum or dermal carotenoids. In addition to adiposity, factors include the amount of fat in the diet, smoking, sunlight exposure, medication and supplement use, and alcohol consumption [5, 9]. More research is needed to determine if race or dermal pigmentation influences dermal carotenoid status [9]. Our final models did not include variables to account for race, smoking, alcohol consumption, medication use, or supplement use because they did not meaningfully impact the associations. It is possible that race and smoking did not affect our models because the large majority of our participants were white and nonsmokers. Although the majority of our participants and more than half of women in the US [52] report taking dietary supplements and nearly half of our sample and half of women in the US report consuming alcohol [53], including these as covariates did not strengthen our models. Controlling for carotenoid-containing supplement use also did not strengthen models. Misreporting of personal characteristics or dietary intake is another possible reason why self-reported FVI did not relate to serum carotenoids in this study. The potential of social desirability bias cannot be eliminated, especially when data collection is connected to a health-related intervention [54]. However, we sought to limit this by administering our baseline survey online and utilizing established measures of health risk behaviors [55]. Previous studies have shown misreporting to be associated with personal characteristics, with underreporting of total energy and overreporting of FVI to be more prevalent among overweight and obese individuals [56–61]. Additionally, blood and dermal carotenoids do not account for the variability of carotenoid content between different fruit and vegetables in the food group [62].

The external validity of our findings may be limited by the focus on overweight or obese women age 40+ years who were predominantly white. However, the important associations between body composition and blood carotenoids [39–41], as well as previously identified differences in the performance of dietary assessment instruments by race/ethnicity [29, 63], suggest analyses performed separately for different population subgroups may be particularly informative. Future investigations should test these associations in additional demographic groups and with larger samples.

Internal validity can also be challenged by the standards and control materials for laboratories measuring carotenoids using HPLC. In this study, however, we used Craft Technologies, Inc., a laboratory that has been performing this analysis for over 20 years and has accumulated the required standards. Additionally, the laboratory participates in the National Standards and Technology (NIST) quality assurance program that provides evaluation of laboratory performance on biannual blinded samples. While a strength of our study is the availability of multiple direct and indirect measures of carotenoids along with a robust set of covariates, extensive data collection (e.g. seven days of recalls) may contribute to respondent fatigue. If this resulted in only highly motivated participants completing data collection, selection bias could have increased the strength of observed associations. Alternatively, if weary respondents completed the recalls but failed to fully report their intake, the strength of the associations could have been weakened. Nevertheless, complete data from all respondents was essential to conduct this study and contributes to the important and growing evidence on dietary assessment methods.

## Conclusions

This study evaluated multiple non-invasive measurement techniques for estimating carotenoid status in overweight and obese women under four potential data collection scenarios. Dermal carotenoid score was superior to any of the self-reported FVI measures in terms of agreement with serum carotenoids, which makes sense given the shared intrinsic and extrinsic factors that impact blood and dermal carotenoid concentrations [9]. Of note, in this study of overweight and obese women, we found there to be no advantage to multiple 24-h recalls over rapid self-report tools for measuring FVI to characterize carotenoid status, which is important to consider when endeavoring to minimize participant burden and maximize limited resources when using self-report tools. Further, we found that the inclusion of a geographic proxy, age, BMI, and blood cholesterol strengthened our analytic models, providing strong support for researchers to collect these data in future research. Emerging technologies that allow for rapid testing of blood samples in the field (e.g point-of-care tests) could be used to simplify assessment of lipids along with dermal carotenoid measurement.

## Data Availability

The datasets analyzed for the present study are available from the corresponding author on reasonable request.

## Abbreviations

24-h: 24-h
AHA: American Heart Association
AIC: Akaike information criterion
ASA24: Automated Self-Administered 24-Hour Dietary Assessment Tool-2014
FFQ: Food frequency questionnaire
FVI: Fruit and vegetable intake
FVS: All-Day Fruit and Vegetable Screener
kcal: Kilocalories
NCI: National Cancer Institute
NIST: National Standards and Technology
RRS: Resonance Raman spectroscopy
SHHC: Strong Hearts, Healthy Communities
